# Supplementary material for: EnzML: multi-label prediction of enzyme classes using InterPro signatures
Source: BMC Bioinformatics. 2012 Apr 25;13:61. doi: 10.1186/1471-2105-13-61 (PMC3483700; doi:10.1186/1471-2105-13-61)
Supplement: Addtional file 5 — The Java code to format the data files, evaluate and predict. The file enzml_java_code.tar.gz contains the Java code used to format database data to ARFF and XML formats, to execute cross and train-test (jackknife) evaluations and to record evaluation results to database. More information is included in the readme.txt file and the Javadoc files. The code can be used with a MySQL database. To use a different database software, other JDBC drivers might be required. [file 1471-2105-13-61-S5.gz › java_code/enzml2011/doc/uk/ac/ed/inf/taweka/mulan/learn/MulanSerializer.html]

MulanSerializer


---


|  |  |  |
| --- | --- | --- |
| **Overview** \| **Package** \| **Class** \| **Use** \| **Tree** \| **Deprecated** \| **Index** \| **Help** | |  |
| **PREV CLASS**   **NEXT CLASS** | **FRAMES**    **NO FRAMES**     **All Classes** |  |
| SUMMARY: NESTED \| FIELD \| CONSTR \| METHOD | DETAIL: FIELD \| CONSTR \| METHOD |  |


---


## uk.ac.ed.inf.taweka.mulan.learn Class MulanSerializer

```
java.lang.Object
  uk.ac.ed.inf.taweka.mulan.learn.MulanSerializer
```

---

``` public class MulanSerializer extends java.lang.Object ```

Takes an arff file and an algorithm. Builds the trained learner. Saves the
trained classifier to file as a serialized object ('.model' suffix).

**Version:**
:   29 Jun 2010

**Author:**
:   Luna De Ferrari luna.deferrari-at-ed.ac.uk

---

**Constructor Summary**

`MulanSerializer(MulanArffRecord arffRecord,
MulanLearner learner,
java.lang.String directoryForSerializedModel)`


| **Method Summary** | |
| --- | --- |
| `void` | `buildAndSerialize()` |
| `static mulan.classifier.MultiLabelLearnerBase` | `deserialize(java.lang.String fileName)`             Get trained learner from file |
| `java.lang.String` | `formatFileName()` |
| `MulanArffRecord` | `getArffRecord()` |
| `java.lang.String` | `getDirectoryForSerializedModel()` |
| `java.lang.String` | `getFileForSerializedModel()` |
| `MulanLearner` | `getLearner()` |
| `java.lang.String` | `getLogFile()` |
| `static void` | `main(java.lang.String[] args)` |
| `boolean` | `serialize(mulan.classifier.MultiLabelLearnerBase model)` |
| `void` | `setFileForSerializedModel()` |
| `void` | `setLogFile()` |

**Methods inherited from class java.lang.Object**

`equals, getClass, hashCode, notify, notifyAll, toString, wait, wait, wait`

**Constructor Detail**

### MulanSerializer

```
public MulanSerializer(MulanArffRecord arffRecord,
                       MulanLearner learner,
                       java.lang.String directoryForSerializedModel)
```


**Method Detail**

### buildAndSerialize

```
public void buildAndSerialize()
```

---


### formatFileName

```
public java.lang.String formatFileName()
```

---


### getArffRecord

```
public MulanArffRecord getArffRecord()
```

---


### getDirectoryForSerializedModel

```
public java.lang.String getDirectoryForSerializedModel()
```

---


### getFileForSerializedModel

```
public java.lang.String getFileForSerializedModel()
```

---


### getLearner

```
public MulanLearner getLearner()
```

---


### getLogFile

```
public java.lang.String getLogFile()
```

---


### serialize

```
public boolean serialize(mulan.classifier.MultiLabelLearnerBase model)
```

---


### setFileForSerializedModel

```
public void setFileForSerializedModel()
```

---


### setLogFile

```
public void setLogFile()
```

---


### deserialize

```
public static mulan.classifier.MultiLabelLearnerBase deserialize(java.lang.String fileName)
```

:   Get trained learner from file

    :   **Parameters:**: `fileName` - the full file path **Returns:**: a multi-label learner base

---


### main

```
public static void main(java.lang.String[] args)
```


---


|  |  |  |
| --- | --- | --- |
| **Overview** \| **Package** \| **Class** \| **Use** \| **Tree** \| **Deprecated** \| **Index** \| **Help** | |  |
| **PREV CLASS**   **NEXT CLASS** | **FRAMES**    **NO FRAMES**     **All Classes** |  |
| SUMMARY: NESTED \| FIELD \| CONSTR \| METHOD | DETAIL: FIELD \| CONSTR \| METHOD |  |


---
